# The Impact of Improving Suicide Death Classification in South Korea: A Comparison with Japan and Hong Kong

**DOI:** 10.1371/journal.pone.0125730

**Published:** 2015-05-20

**Authors:** Chee Hon Chan, Eric D. Caine, Shu Sen Chang, Won Jin Lee, Eun Shil Cha, Paul Siu Fai Yip

**Affiliations:** 1 Department of Social Work and Social Administration, The University of Hong Kong, Hong Kong Special Administrative Region, China; 2 The Hong Kong Jockey Club Centre for Suicide Research and Prevention, The University of Hong Kong, Hong Kong Special Administrative Region, China; 3 The Injury Control Research Center for Suicide Prevention, Department of Psychiatry, University of Rochester Medical Center, Rochester, United States of America; 4 Institute of Health Policy and Management, and Department of Public Health, College of Public Health, National Taiwan University, Taipei, Taiwan; 5 Department of Preventive Medicine, Korea University College of Medicine, Korea University, Seoul, South Korea; University of Vienna, AUSTRIA

## Abstract

**Introduction:**

The suicide rate of South Korea has increased dramatically during the past decades, as opposed to steadily decreasing trends in Japan and Hong Kong. Although the recent increase of suicide in South Korea may be related to changing socioeconomic conditions and other contextual factors, it may also reflect, in part, a reduction of misidentified suicide cases due to improving classification of manner of death.

**Method:**

We compared the annual proportional change of suicide, undetermined death, and accidental death from South Korea with those of Japan and Hong Kong from 1992 to 2011; a greater proportional change of the manner-of-death categories during the period is indicative of a relatively less stable registration and hence a greater potential for misclassification bias on reported suicide trends. Subgroup analyses stratifying the deaths by methods were also conducted. To estimate the impact, the age-standardized rates of these three death categories in each site were calculated.

**Results:**

We found that, during the 20-year observation period, the proportional change of suicide, undetermined death, and accidental death in South Korea was significantly greater than Japan and Hong Kong. Similar observations were made in subgroup analyses. While death rates of the three manners in Japan and Hong Kong generally moved in a parallel fashion, the increase of suicide in South Korea occurred concomitantly with a significant reduction of its accidental death rate. 43% of the increase in suicides could be attributed to the decrease in accidental deaths, while 57% of the increase could be due to fundamental causes.

**Conclusion:**

Our data suggest that, during the mid-1990s and after, the increasing burden of suicide in South Korea initially was masked, in part, by misclassification. Thus, the later apparently rapid increase of suicides reflected steadily improving classification of manner of death, as well as a more fundamental increase in the suicide rate.

## Introduction

Suicide rates in many countries are underestimated; the degree of such underestimation varies substantially by nations [1–3]. In Asia, social stigma has been associated with non-disclosure of suicidal intent, and it remains illegal in some countries (e.g. Singapore, Malaysia and India) [4]. Undercounting of suicides also relates to poor case ascertainment, inadequate investigation, incomplete reporting and variable classification due to the background, attitudes, training, and rigor of medical examiners or coroners, who may be appointed or elected officials, and whose offices may not be certified by outside professional organizations [1–3]. Deaths categorized as “undetermined” with respect to intent, and those labeled as “accidental” or “unintentional” (especially, poisoning and drowning) are likely reservoirs of the misclassified suicides [2,5,6]. Reports have highlighted the issue of suicide underestimation in many Asian nations (including South Korea, Japan, and Hong Kong), pointing to both those with undetermined intent as well as so-called “accidents” [7–12].

Several authors have proposed that change in death registration processes would affect the degree of underestimation in the reported suicide statistics (i.e., misclassification hypothesis) [13,14]. One illustration was from a recent examination showing the reduced diagnostic accuracy (autopsy-rate) in 35 European countries from 1979 to 2007 contributed to their declining suicide trends even after adjusting for socioeconomic factors [14].

In South Korea where medical certification of death is not mandatory, the varying effort for cause-of-death ascertainment as well as changing attitudes in disclosure may affect the completeness of the reported suicide statistics. In earlier years (e.g. before 2000), Korean suicide statistics has been considered as less reliable [7]. A World Health Organization (WHO) report evaluating the death statistics of 115 countries from 1990–1999 graded Korean’s death statistics with moderate quality (due to incomplete death registration and greater number of ill-defined deaths); it ranked lower than the Japanese’s, falling in the high quality category [15]. However, since 1999, the death registration in South Korea has undergone various modifications. According to Statistics Korea, they have extended its linkages of the registered death statistics to several other administrative datasets (e.g. National Health Insurance data, and Police Report for Incident or Injury). Reports have suggested the quality of the death statistics has improved over the years and the accuracy of identifying suicides has improved [7,11]. Thus, changes of death registration in South Korea may have served to reduce erroneous misclassifications, and in the process, added to reported annual suicide statistics. In Japan and Hong Kong, where all deaths must undergo formal examination conducted by medical examiners or coroners supported by trained pathologists, the death registration has been fairly consistent in the past two decades.

Concomitantly, the trend of suicides in South Korea during the past two decades also changed more dramatically than those of Japan and Hong Kong. Based on the OECD heath statistics, suicide in South Korea during the mid-90s was comparable with Japan, but it surpassed Japan during the early years of the 21^st^ century and exceeded 30 per 100,000 by 2009 [16]. A recent report from the WHO also showed that the increase of suicide in South Korea from 2000 to 2012 was more than two-fold, contrasting with recent decreasing suicide trends in many Southeast Asian nations (including Japan and Hong Kong) [17]. While this rapid increase of suicide may be related to various changes of socioeconomic conditions and other national contextual factors [18,19], it also may reflect improvements of classifying manner of death. To explore this issue, we first analyzed the proportional change of deaths across suicide, undetermined deaths, and accidental deaths in South Korea from 1992 to 2011, and compared these with Japan and Hong Kong. In this context, we posited that a greater proportional change of deaths across manner of death is indicative of a relatively less stable registration and hence may have a greater misclassification bias on the current suicide trends. To estimate the impact, the age-standardized rates of the three manners for all three sites were calculated.

## Methods

### Data collection

We collected mortality and the population statistics of South Korea, Japan, and Hong Kong for the years 1992–2011. For South Korea, individual-level death data and population statistics were obtained from the Statistics Korea. For Japan, deaths aggregated by 5-year age groups and population estimates were extracted from the WHO mortality database. Data in the WHO mortality dataset were collected from the civil registration system in its respective nation; in the case of Japan, it was from the Ministry of Health. The WHO dataset also included death statistics of South Korea and it was sourced from Statistics Korea. For Hong Kong, individual-level registered deaths and population statistics were from the Census and Statistics department (C&SD). Given the nature of the information used, based on analyses of de-identified data and population statistics, this study was exempted from ethical review by the Human Research Ethics Committee for Non-Clinical Faculties, The University of Hong Kong.

### Data analyses

Injury-related deaths of South Korea, Japan, and Hong Kong were extracted for analyses (i.e., coded E800–E999 ICD-9^th^ and V01—Y98 in the ICD-10^th^). In particular, we examined three categories of the injury-related deaths, namely: suicide (deaths with codes X60–X84 in ICD-10^th^ and E950–E959 in ICD-9^th^; abbreviated as S), undetermined death (deaths with codes of Y10–Y34 in ICD-10^th^ and E980–E989 in ICD-9^th^; abbreviated as U) and accidental death (abbreviated as A). Accidents include deaths that previously have been noted to have a high likelihood for involving misclassified suicides, such as accidental poisoning (X40–X49 in ICD-10^th^ and E950–E869 in ICD-9^th^), drowning (W65–W74 in ICD-10^th^ and E910 in ICD-9^th^), suffocation (W75, W76, W83, W84 in ICD-10^th^ and E913 in ICD-9^th^), and falling (W00–W19 in ICD-10^th^ and E800–E888 in ICD-9^th^) [3,10,11,20]. For Japan, we included the data only from 1995 to 2011 in this analysis, given that method-specific accidental death data were not available in the WHO mortality database before 1995. Also, although the WHO mortality database consisted death statistics of South Korea, it however did not have a full spectrum of method-specific accidental deaths before 1995; thus we relied on data obtained directly from Statistics Korea. A quick exploration showed the number of deaths across the three manner of deaths from these two sources were almost identical (S1 Table).

Based on the number of suicides (S), undetermined (U) and accidental (A) deaths, we estimated the proportional change of each category (S, U, A) from 1992–2011, reflecting changes in registration. In the context of this study, we posited that greater proportional change during the study period for each death category would be indicative of a relatively less stable registration system. Hence, changes in death rates of the nations over the study period would be more affected by the change of manner of death determination. Sub-group analyses further stratifying the deaths by the four methods (i.e. hanging/suffocation, poisoning, falling, and drowning) were also conducted. To visualize the potential effect of the changing death classification, we estimated the age-standardized rates of these three deaths categories for each nation in the same study period (1992–2011). To make our comparisons meaningful, we used a 5-year age interval standardization technique with the World Standard Population 2001 to adjust for demographic and interval differences within and between our three sites.

## Results

**Fig 1:**
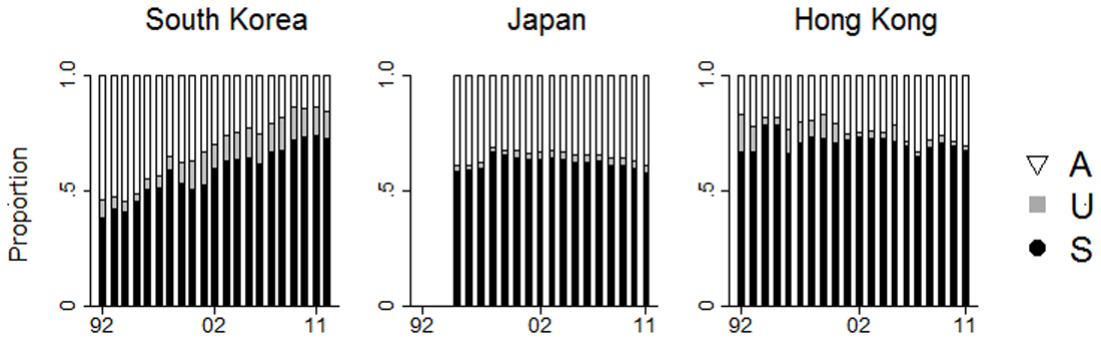
Annual proportional change of suicides, undetermined deaths, and accidental deaths in South Korea, Japan, and Hong Kong from 1992 to 2011.

The total number of suicides during the study period (1992–2011) for South Korea, Japan, and Hong Kong were 171,616, 489,276, and 18,181, respectively. Undetermined deaths were reported as 20,052, 32,888, 1,476, respectively, and accidental deaths were 94,147, 277,903, and 6,158, respectively. The population of South Korea, Japan, and Hong Kong all increased gradually over the study period (South Korea: from 44.8M in 1992 to 50.1 M in 2011; Japan: from 124.3M in 1995 to 126.2M in 2011; Hong Kong: from 5.8M in 1992 to 7.1 M in 2011). Table 1 summarizes the proportion of deaths and the age-standardized rate of suicide, undetermined and accidental deaths in South Korea, Japan, and Hong Kong in 1992 and 2011. The annual proportional change of the death categories in all three sites during the study period is shown in Fig 1. Compared to Japan and Hong Kong, the proportional change of the death categories in South Korea during the 20-year study period was dramatic. Specifically, the substantial proportional increase of suicide over the period was counterbalanced by the gradual proportional decrease of accidental death (Suicide: Δ = .38; Accident: Δ = -.43). Also, there was an increased proportion of undetermined death in South Korea but the magnitude was far lesser than the suicide (Δ = .05). In Japan and Hong Kong, proportional changes of the three death categories during the study period were relatively small. Greater instability across the death categories was noted in Hong Kong as compared to Japan, but it is likely due to a smaller size of the community and year-to-year fluctuations, rather than the steady upward increase in the proportion of suicides in South Korea.

**Table 1:** The age-standardized rate and proportional change of suicides, undetermined deaths, and accidental deaths from 1992 to 2011.

	Age-standardized rate			Proportion across the three manners		
	1992	2011	Δ	1992	2011	Δ
***South Korea***						
Suicide	3650 (8.2)	15942 (26.0)	12292 (17.8)	.34	.72	.38
Undetermined death	758 (1.7)	2647 (3.4)	1889 (1.7)	.07	.12	.05
Accident	6173 (12.9)	3443 (5.2)	-2730 (-7.7)	.58	.16	-.43
***Japan*** ^a^						
Suicide	18898 (13.4)	26999 (18.2)	8101 (4.8)	.59	.58	-.01
Undetermined death	730 (6.9)	1383 (5.7)	653 (-1.2)	.02	.03	.01
Accident	12630 (0.8)	18176 (1.3)	5546 (0.5)	.39	.39	.00
***Hong Kong***						
Suicide	716 (11.7)	847 (9.2)	131 (-2.5)	.67	.67	.00
Undetermined death	186 (2.8)	26 (0.3)	-160 (-2.5)	.17	.02	-.15
Accident	172 (3.1)	388 (3.7)	216 (0.6)	.16	.31	.15

^a^ Since method-specific data of accidental death in 1992 to 1994 is not available in WHO mortality dataset, the death data of 1995 is used for the analysis.

**Fig 2:**
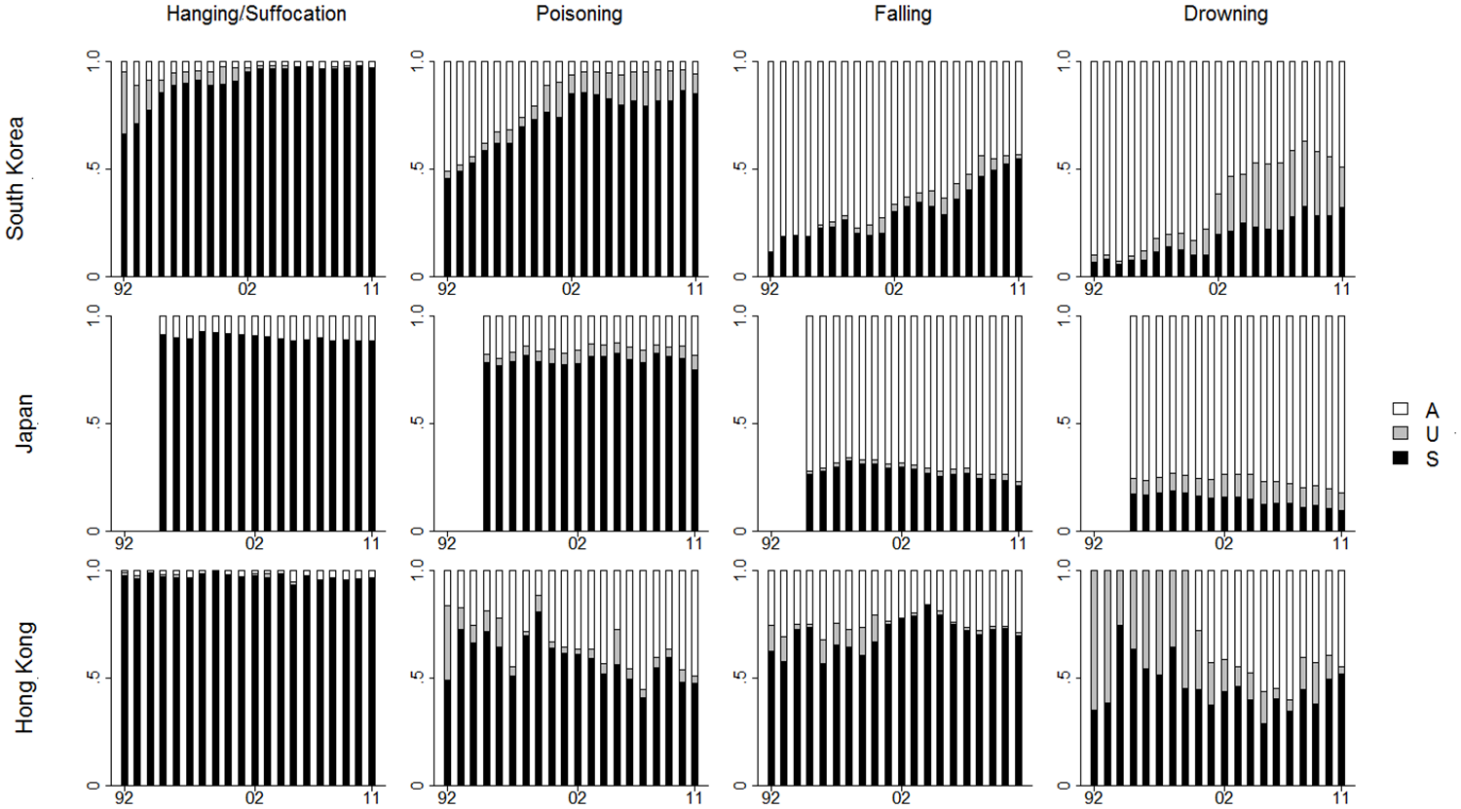
Annual proportional change of suicide, undetermined death, and accident in South Korea, Japan, and Hong Kong stratified by methods from 1992 to 2011.

Fig 2 details the annual proportional change of the three death categories during the study period related to specific methods. The results show great similarity to the aggregated analysis. Specifically, the pattern involving increasing proportion of suicides and reduced accidental deaths in South Korea was evident across all methods; the largest proportional increase was noted from poisoning (Suicide:Δ = .40; Undetermined death: Δ = .05; Accidental death: Δ = -.45). In Japan, despite the slight proportional decrease of suicide for falling and drowning, the overall proportional changes of the death categories across all methods were still comparatively small. For Hong Kong, a further stratification of the deaths, causing fewer cases in each method, resulted in even greater instability across death categories over the study period; but a change of registration pattern was evident for drowning in 2000 (i.e., increased use of accident). For all three sites, changes of the accidental and undetermined deaths were comparatively smaller for hanging/suffocation than other methods; given that hanging and other forms of suffocation typically require implements and preparation, it is unsurprising that there were few cases determined to be accidental or undetermined.

**Fig 3:**
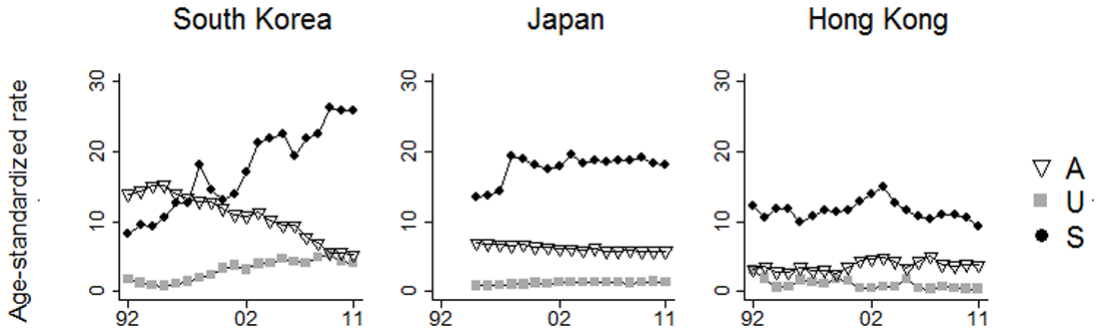
Age-standardized rates of suicide, undetermined death, and accident in South Korea, Japan, and Hong Kong from 1992 to 2011.

Trends of the age-standardized death rates in South Korea, Japan, and Hong Kong from 1992–2011 are shown in Fig 3 (the method-specific age-standardized death rates of all three sites are shown in S1 Fig). In South Korea, the increase in suicide was more than threefold (17.8 per 100,000), as opposed to a much smaller change in Japan (an increase of 4.8 per 100,000) and Hong Kong (a decrease of 2.5 per 100,000). On the other hand, accidents in South Korea conversely showed a two-fold reduction (a decrease of 7.7 per 100,000), contrasting to a slight increase in Japan (an increase of 0.5 per 100,000) and Hong Kong (an increase of 0.6 per 100,000). Undetermined deaths in South Korea increased from 1.7 per 100,000 in 1992 to 3.4 per 100,000 in 2011, contrasting to the decreasing trends in Japan and Hong Kong (Japan: -1.2 per 100,000; Hong Kong: -2.5 per 100,000). Given the rigorous efforts to improve manner of death classification in South Korea, we can estimate that 43% (7.7/17.8 = 0.432) of the increase in suicides was attributable to the decrease in accidental deaths (i.e. registration change), and that 57% ((17.8–7.7)/17.8 = 0.568) of the increase could have been due to other reasons (i.e. fundamental contextual changes).

## Discussion

South Korea, Japan, and Hong Kong experienced rapid economic development during recent decades, as well as having many common cultural and social values (e.g. Confucianism) [21]. Their suicide surveillance systems share many features [4,7]. Hence, trends of suicide, particularly involving Japan and South Korea, often have been compared [22,23]. The results of our analyses emphasize the need for caution when making such comparisons, given that our findings strongly indicate that South Korea has experienced striking changes in death classification. Our results also echo with studies highlighting the discrepancy of the completeness and the quality of the death data (percent of death with uncertain cause), particularly between Japan and South Korea [15,24].

While changes in demographic, social, and economic conditions in South Korea contributed to the recent rise in its suicide rates [18,19], our results indicate that the apparent rapid increase during the past two decades can be attributed, in part, to the growing rigor of post-mortem examination and classification of manner of death, thus shifting a substantial number of cases over the study period from “accidental” to suicide—especially involving methods where determining intent is more ambiguous (e.g., poisoning). Our results point to the value of considering potential misclassified suicides (i.e., undetermined and accidental deaths) when investigating the effects of socioeconomic factors on death trends [9,25].

Of course, this change does not mitigate the heavy burden posed by all causes of potentially preventable deaths, including suicides, accidents, and undetermined deaths, but it does underscore the need for accurate surveillance data in order to inform the public and policy makers about nationally important priorities. In essence, recognition of the rising tide of suicides in South Korea may have been delayed by initially underestimating the national burden.

We recognize clear limits to the conclusions that can be drawn from this kind of population level study. For example, accidental deaths have decreased in South Korea, Japan, and Hong Kong during recent decades, attributable in great part to reduced traffic related deaths [9,26–28]. Given the relatively small number of suicides that likely are to be coded as traffic-related, as well as government policies designed to actively reduce such injuries [29], we chose not to explore whether these may have been a source of misclassification, preferring to focus on those methods with a higher probability of misclassification [3,10,11,20]. Determining the exact extent of misclassification always will confront uncertainties, even when conducting individual in-depth examinations. We estimated that 43% of the apparent increase in suicides reflected improving accuracy, and 57% pointed to an actual increase in the number of deaths above the gain from the improving system. While these cannot be viewed as exact figures, they provide a clear indication of the apparent magnitude of the impact derived from enhancing the quality of death determinations, with greater physician participation and care [7,30]. These results do not serve to lessen the gravity of the suicide-related challenge in South Korea; rather they suggest that sounding the alarm may have been delayed by inadequate surveillance methods.

It is important to underscore that this type of problem is not unique to South Korea. The rate of so-called “accidental deaths” in the United States (US) from poisoning with prescription medications, illicit opiates, and other drugs, has skyrocketed in recent years [31]. Given the highly inconsistent nature of manner of death classification in the US, with both state-to-state and in-state variability, it is highly plausible that a problem similar to what we have found in South Korea exists in the US.

## Conclusion

The national data available for South Korea amply demonstrate that, at this time, suicide represents a major public health challenge. Our results suggest, however, that this problem may have been masked for many years by the misclassification of many suicides as either accidents or undetermined deaths. In turn, the apparent rapidity in the rate of the rise in South Korea’s suicide rates may have in part reflected the procedural changes that led to more accurate appraisals of manner of death. Whatever the reason, the current heavy burden in death from suicide creates urgency for developing and disseminating effective prevention measures.

Accurately determining manner of death is a keystone for evaluating the impact of any prevention initiatives. Better classification of suicide in South Korea likely would have provided a warning regarding the rising tide of suicide, and allowed an earlier sounding of the alarm.

## Supporting Information

S1 Fig Age-standardized rates of suicide, undetermined death, and accident in South Korea, Japan, and Hong Kong from 1992 to 2011 stratified by methods. (TIF)

S1 Table Number of deaths and age-standardized death rates of suicide, undetermined death, and accident of South Korea in 1992 and 2011 (sourcing from WHO mortality data and Statistic Korea). (DOCX)
