# Supplementary figures and images for: The Impact of Improving Suicide Death Classification in South Korea: A Comparison with Japan and Hong Kong
Source: PLoS One. 2015 May 20;10(5):e0125730. doi: 10.1371/journal.pone.0125730 (PMC4439106; doi:10.1371/journal.pone.0125730)

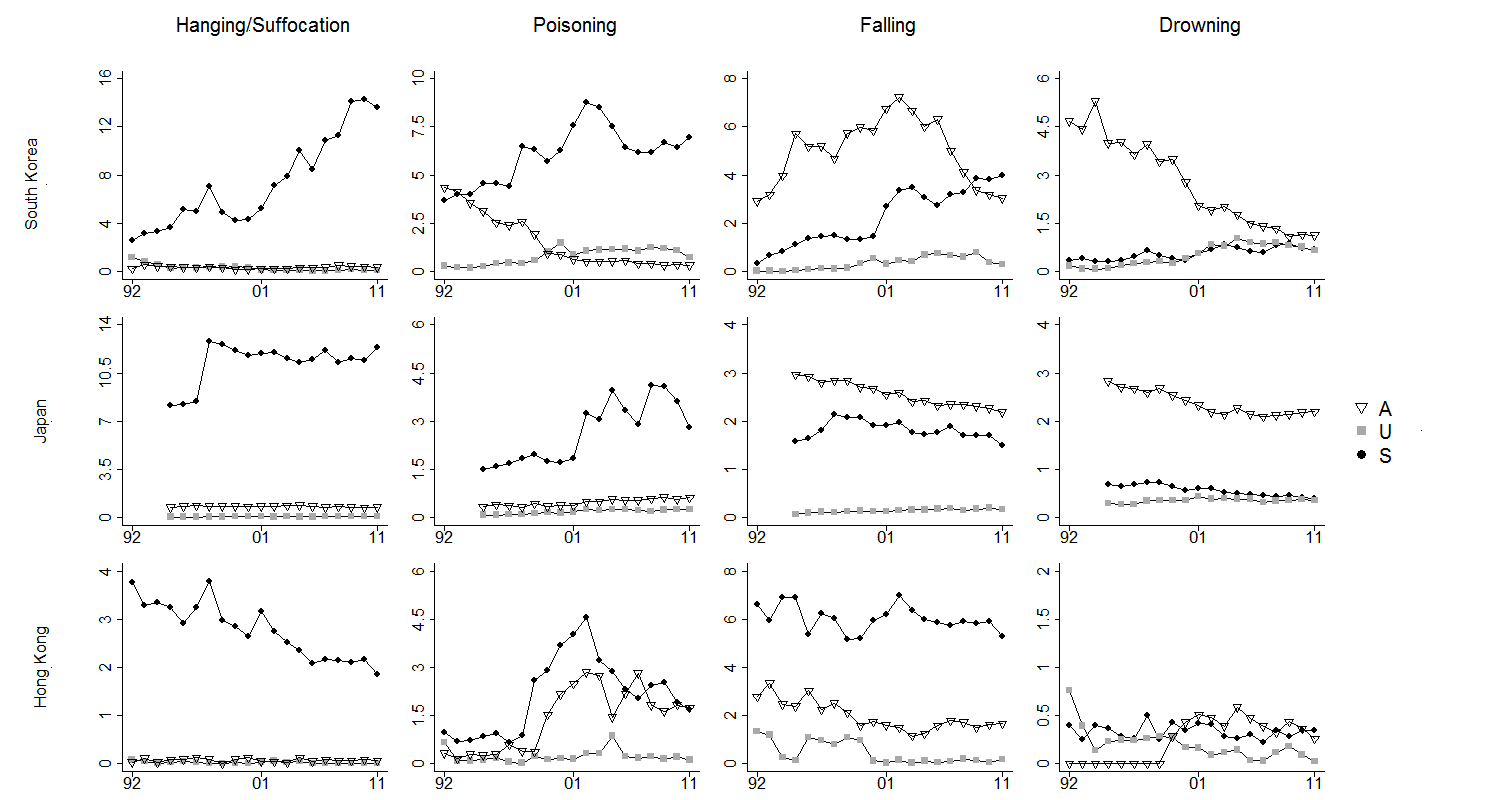

Supplement: S1 Fig — (TIF) [file pone.0125730.s001.tif]
