# Supplementary material for: The Impact of Improving Suicide Death Classification in South Korea: A Comparison with Japan and Hong Kong
Source: PLoS One. 2015 May 20;10(5):e0125730. doi: 10.1371/journal.pone.0125730 (PMC4439106; doi:10.1371/journal.pone.0125730)
Supplement: S1 Table — (DOCX) [file pone.0125730.s002.docx]

**Supporting Information**

S1 Table: Number of deaths and age-standardized death rates of suicide, undetermined death, and accident of South Korea in 1992 and 2011 (sourcing from WHO mortality data and Statistic Korea)

|  | 1992 | 2011 |
| --- | --- | --- |
| ***Statistic Korea*** |  |  |
| Suicide | 3650 (8.2) | 15942 (26.0) |
| Undetermined death | 758 (1.7) | 2647 (3.4) |
| Accident | 6173 (12.9) | 3443 (5.2) |
| ***WHO*** |  |  |
| Suicide | 3533 (8.2) | 15901 (25.9) |
| Undetermined death | 741 (1.8) | 2623 (3.4) |
| Accident | - | 3078 (5.1) |
